# Efficacy of 5-fluorouracil (5-FU) and low molecular weight heparin (LMWH) in high-risk pediatric retinal detachment; randomized clinical trial

**DOI:** 10.1186/s12886-024-03362-4

**Published:** 2024-03-04

**Authors:** Mohamed Nasr, Ahmed Abdelhadi, Amr Bessa, Tamer Moussa Ibrahim

**Affiliations:** Department of Ophthalmology, Faculty of Medicine, Alexandria University, Alexandria, Egypt

**Keywords:** Pediatric retinal detachment, Proliferative vitreoretinopathy, 5-fluorouracil, Low molecular weight heparin, Recurrent retinal detachment

## Abstract

**Background:**

Pediatric rhegmatogenous retinal detachments (PRRDs) are complex, rare occurrences and are often related to trauma or congenital abnormalities. Children often do not recognize or report symptoms of retinal detachment. Thus at presentation, PRRD is typically advanced often with macular involvement, proliferative vitreoretinopathy (PVR), chronic duration, and poor visual acuity. Because 5-FU and LMWH are effective in different aspects in the PVR process, it was believed that a syngergistic approach to the prevention of PVR would be advantageous.

**Methods:**

After informed consent, children under 14 years of age with high-risk PRRD underwent pars plana vitrectomy and silicone oil injection with scleral buckle divided into 2 groups in prospective randomized trial. Group A received intraoperative infusion of 5-FU (200 µg/ml) and LMWH (5 IU/ml), group B received infusion of normal saline. Primary outcome was occurrence of recurrent PRRD within 12 weeks, secondary outcomes were occurrence of PVR, best corrected visual acuity (BCVA), number and timing of secondary procedures within 12 weeks.

**Results:**

The study included 42 eyes of 41 patients, 21 in group A and 21 in group B, the duration of PRRD ranged from 0.5 to 7 months in group A and 0.25-5 months in group B.The rate of recurrent PRRD was higher in group B 33% compared to 19% in group A (*p* = 0.292). The mean timing of occurrence of recurrent PRRD was 9.5 ± 5 weeks in group A compared to 2.86 ± 2.41 weeks in group B (*p* = 0.042), more patients in group B ended up with more advanced PVR (*p* = 0.038), BCVA was hand movement (HM) only in all cases preoperatively and improved to HM-0.3 Snellen in group A compared to light perception (PL)-0.1Snellen in group B (*p* = 0.035), there was no difference in any of secondary procedures but with later timing in group A 9.71 ± 3.73 weeks than in group B 4.0 ± 2.83 weeks (*p* = 0.042).

**Conclusion:**

This study concluded that the use of the 5-FU and LMWH combination in high risk PRRD resulted in lower rate of postoperative PVR, later recurrence of PRRD and better final BCVA.

**Trial registration number:**

Registry: clinicaltrials.gov PRS NCT06166914 date of initial release 4/12/2023. Unique Protocol ID: 9,163,209 date 21/10/2021. Retrospectively registered

## Background

Pediatric rhegmatogenous retinal detachments (PRRDs) are complex, rare occurrences and are often related to trauma, congenital abnormalities, genetic syndromes, high myopia, and prior ocular surgery. This is in contrast to the most common causes of rhegmatogenous retinal detachment (RRD) in adults, which is related to retinal breaks that develop during a posterior vitreous detachment (PVD).

In the general population, RRD has an annual incidence of approximately 10 cases per 100,000 population [1]. PRRD is much less common with an annual incidence of only 0.38 to 0.69 per 100,000 in the population under age 20 years [2] but makes up 3–13% of all retinal detachments [3–5]. The average age at presentation of PRRD is between 7 and 13 years of age. Trauma is the most common cause of PRRD and accounts for about 40% of all RRD in children compared to 11% of RRD in the adult population. There is a significant male preponderance of PRRD, especially in trauma-related cases (up to 80%), but even in nontraumatic PRRD [6, 7].

Children often do not recognize or report symptoms of retinal detachment. Thus at presentation, the pediatric pathology is typically advanced often with macular involvement, chronic duration, and poor visual acuity. Additionally, pediatric patients also have much higher rates of PVR, between 30% and 60%, on presentation and with 10–20% with at least PVR grade C [2, 6, 7].

For all of these reasons, visual potential after repair of PRRD may be limited even after successful surgical repair because of chronicity and the effects of deprivation amblyopia [8–10].

The single-procedure anatomical success rates range from approximately 50–90% [11] often due to late presentation, while the reported overall anatomic success rate of surgical repair of pediatric RRDs ranges from 74.9 to 80% [8, 10] with PVR being the most common cause of failure of surgical repair.

The devastating effect of PVR on visual prognosis and the expense and difficulty of multiple surgeries has led to sustained efforts to find pharmacologic therapies that may decrease the risk and recurrence after retinal detachment surgery. Laboratory and clinical studies suggest that pharmacologic adjuvant therapy can modify the proliferative disease process and improve the success of surgery. There are a number of studies showing a potential benefit of a variety of pharmacologic interventions, including retinoic acid [12–15], dexamethasone [16, 17], colchicine [18, 19], paclitaxel (Taxol) [20, 21],, daunorubicin [22, 23], mitomycin C [24]. However, none of these regimens are in routine clinical use.

5-fluorouracil (5-FU) has been shown to be effective in reducing the rate of PVR in animal models [25, 26]. Toxicity studies using either single or multiple intravitreal injections of 5-FU produced no morphologic or electrophysiologic changes in the rabbit retina at low dosages [27]. 

Other than being used alone, its use in combination with other drugs have been investigated. 5-FU in combination with triamcinolone or low-molecular-weight-heparin (LMWH) has been injected into rabbit models of PVR [28, 29]. Results showed significant reduction in terms of RRD and severity of the PVR [28, 29]. As far as toxicity is concerned, no demonstrable damage was seen in the animal eyes.

Low molecular weight heparin (LMWH) is a multipotential drug useful in the treatment of PVR [30]. Animal work has shown that LMWH is effective in reducing the rate of retinal detachment [30] and that it produces no toxic effects in the rabbit eye when infused using a dose of 5 IU/ml [31].

Although the efficacy of both chemicals was tested in multiple previous studies on adults with varying results, as far as the authors are aware of, they were not tested in pediatric retinal detachment which is usually associated with high incidence of PVR.

## Methods

All methods were carried out in accordance with relevant guidelines and regulations.

### Subjects

The study included 42 eyes of children under 14 years of age suffering from high risk rhegmatogenous retinal detachment (RRD) undergoing pars plana vitrectomy (PPV) and silicone oil injection with scleral buckle divided into 2 groups. Informed consent from parents or guardians was taken after thorough explanation of the potential success and complication rates.

Children under 14 years of age with RRD undergoing primary repair were included, as well as those with preoperative PVR grade B or higher and those with high risk RRD: uveitis; large, giant, or multiple tears; vitreous hemorrhage; preoperative choroidal detachments; aphakia; and large detachments involving greater than two quadrants of the eye.

Children with RRD related to penetrating ocular trauma involving the posterior segment were excluded, as well as those with previous RD repair surgery, uncontrolled glaucoma or other concomitant ocular morbidities, patients with bleeding diathesis, hepatic and renal failure, corneal opacity sufficient to impair surgical view, no light perception vision or inability to complete follow-up.

### Study design

The study was conducted as single-centered, randomized clinical trial that comprised children under 14 years old undergoing 23-guage (G) PPV and silicone oil injection with scleral buckle to study the efficacy and safety of using adjuvant 5-FU and LMWH intraoperative infusion.

All surgeries were performed under general anesthesia. A standard 3-port 23-G PPV was performed, along with lensectomy for lens opacity or the management of anterior PVR.

Elimination of traction sufficient to allow retinal reattachment was achieved by epiretinal membrane peeling or relaxing retinotomy and retinectomy. Retinopexy was applied to treat retinal breaks using endolaser and/or cryotherapy. A scleral buckle or encircling band was used in all cases. Silicone oil was used for internal tamponade. A 6 o’clock iridotomy was done for aphakic silicone filled eyes. Proper postoperative positioning was advised in all the patients.

Qualified patients were randomized into 2 groups before surgery. Group A received intraoperative intraocular infusion of 5-FU (200 µg/ml) and LMWH (5 IU/ml) through the irrigation bottle, group B received intraocular infusion of normal saline, randomization was performed on consecutive patients enrolled into the study within specified time period. Patients were evaluated preoperatively, at 1 day and at 6 and 12 weeks postoperatively using one or more of best corrected visual acuity (BCVA) assessment, slit-lamp examination, indirect ophthalmoscopy examination, fundus photography, in awake patient or during examination under anesthesia.

### Outcome measures and follow-up

The primary objective of the trial was to investigate whether the incidence of recurrent RD due to PVR can be reduced in high-risk eyes by intraoperative adjuvant therapy with 5-FU and LMWH. The secondary objective was to investigate whether intraoperative adjuvant therapy with 5-FU and LMWH affected postoperative outcome parameters and postoperative course in high-risk patients with RRD.

***Primary endpoint***:

1. Recurrent RD due to PVR within 12 weeks.

***Secondary endpoints***:

1. Any grade or degree of PVR at 6 and 12 weeks in accordance with the updated classification of PVR of the Retina Society (1991).
2. BCVA measured by Landolt’s broken ring or illiterate E charts within 6 and 12 weeks in cooperative children.
3. Retinal re-attachment after primary intervention (yes/no) within 6 and 12 weeks.
4. Number and extent of surgical procedures necessary to achieve retinal re-attachment within 12 weeks.

### Statistics

Data were fed to the computer and analyzed using IBM SPSS software package version 20.0. (Armonk, NY: IBM Corp). Categorical data were represented as numbers and percentages. Chi-square test was applied to compare between two groups. Alternatively, Fisher Exact or Monte Carlo correction test was applied when more than 20% of the cells have expected count less than 5. For continuous data, they were tested for normality by the Shapiro-Wilk test. Quantitative data were expressed as range (minimum and maximum), mean, standard deviation and median. Student t-test was used to compare two groups for normally distributed quantitative variables. On the other hand, Mann Whitney test was used to compare two groups for not normally distributed quantitative variables. Wilcoxon signed ranks test was used for abnormally distributed quantitative variables, to compare between two periods.

## Results

### Peroperative characteristics of the 2 studied groups

The study included 42 eyes of 41 patients enrolled from September 2021 till September 2022, 21 in group A and 21 in group B, males were more than females (14 to 7 in group A and 13 to 8 in group B) and the median age was 10 years in group A to 8 years in group B but the differences were not statistically significant. Table (1).

There were no statistically significant differences between both groups regarding the assumed high risk ocular findings for PVR which included the presence of multiple breaks, large or giant retinal tears, vitreous or choroidal hemorrhage or extensive retinal detachment of 2 or more quadrants. Table (1).

The duration of RD ranged from 0.5 to 7 months with median of 2 months in group A and from 0.25 to 5 months with median of 2 months in group B, however, there was no statistically significant difference between the 2 groups. Table (1).

The duration of surgery which might be related to exposure time to adjuvants ranged from 30 to 90 min with mean of 64.05 ± 18.68 min in group A and from 30 to 80 min with mean of 57.14 ± 18.0 in group B, however there was no statistically significant difference between the 2 groups. Table (1).

**Table 1:** Comparison between peroperative characteristics of the two studied groups

Gender	Group A (*n* = 21)		Group B (*n* = 21)		Test	p
	No.	%	No.	%		
• Male	14	66.7	13	61.9	χ^2^= 0.104	0.747
• Female	7	33.3	8	38.1		
**Age (years)**	**Group A (** ***n*** ** = 21)**		**Group B (** ***n*** ** = 21)**			
• Min.– Max.	0.83–14.0		3.67–14.0		t = 1.206	0.235
• Mean ± SD.	10.21 ± 3.39		9.0 ± 3.09			
**PVR risk factors**	**Group A (** ***n*** ** = 21)**		**Group B (** ***n*** ** = 21)**			
	**No.**	**%**	**No.**	**%**	**χ** ^**2**^	**p**
• Multiple breaks	5	23.8	3	16.7	0.303	^FE^*p*=0.702
• Large or GRT	10	47.6	4	22.2	2.717	0.099
• Vitreous hemorrhage	5	23.8	5	27.8	0.080	^FE^*p*=1.000
• Choroidal hemorrhage	4	19.0	1	5.6	1.579	^FE^*p*=0.349
• Extensive RD	18	85.7	14	77.8	0.415	^FE^*p*=0.682
**Duration of RD (months)**	**Group A (** ***n*** ** = 16)**		**Group B (** ***n*** ** = 16)**		**U**	**p**
• Min.– Max.	0.50–7.0		0.25–5.0		101.5	0.323
• Mean ± SD.	2.56 ± 1.96		1.85 ± 1.36			
**Duration of surgery (min.)**	**Group A (** ***n*** ** = 21)**		**Group B (** ***n*** ** = 21)**		**U**	**p**
• Min.– Max.	30.0–90.0		30.0–80.0		176.0	0.260
• Mean ± SD.	64.05 ± 18.68		57.14 ± 18.0			

SD: standard deviation t: student t-test χ^2^: Chi square test

p: p value for comparing between the two studied groups FE: Fisher Exact U: Mann Whitney test

### Post-operative outcomes

The rate of recurrent RD within 12 weeks after surgery was higher in group B 33% (7 of 21) compared to 19% (4 of 21) in group A, however, this difference was not statistically significant. Nevertheless, the mean timing of occurrence of recurrent RD was 9.5 ± 5 weeks in group A compared to 2.86 ± 2.41 weeks in group B and this difference was statistically significant. Table (2).

Interestingly, the proportion of patients with preoperatively more advanced PVR grades was higher in group A than in group B, although this difference was not statistically significant, on the contrary, this was reversed at 12 weeks postoperatively, where more patients in group B ended up with more advanced PVR than in group A and this difference was statistically significant. (*p* = 0.038) Table (2).

Best corrected visual acuity (BCVA) was hand movement only in all cases of both groups preoperatively and improved to HM-0.3 in group A compared to PL-0.1 in group B at 12 weeks postoperatively, with more patients achieving statistically significant better final BCVA in group A than in group B. Table (2).

Secondary surgical procedures were required at or before 12 weeks of primary surgery due to multiple causes including recurrence of PVR related RD, silicone oil induced elevations of intraocular pressure (IOP), development of significant cataract obscuring posterior pole view or other visual requirements, procedures ranged from silicone oil removal (SOR) + gas tamponade, SOR + management of PVR with SO reinjection with or without phacoemulsification, SOR + relaxing retinectomies + SO reinjection and SOR only. Statistically, there were no difference between the 2 groups in any of needed procedures but there was statistically significant difference in the timing of needed procedures which was 9.71 ± 3.73 weeks in group A compared to 4.0 ± 2.83 weeks in group B. Table (2).

**Table 2:** Comparison between the two studied groups according to occurrence of recurrent RD - PVR– BCVA - secondary procedures at 12 week

Recurrent RD 12 week	Group A (*n* = 21)		Group B (*n* = 21)		Test	p
	No.	%	No.	%		
• No	17	81.0	14	66.7	χ^2^= 1.109	0.292
• Yes	4	19.0	7	33.3		
**Site of RD**					χ^2^= 1.061	^FE^p= 0.545
• Inferior	1	25.0	4	57.1		
• Total/ Near total	3	75.0	3	42.9		
**Time weeks**					U = 3.00^*^	0.042^*^
• Min.– Max.	2.0–12.0		1.0–6.0			
• Mean ± SD.	9.50 ± 5.0		2.86 ± 2.41			
**PVR**	**Group A (** ***n*** ** = 20)**		**Group B (** ***n*** ** = 20)**		**χ** ^**2**^	^**MC**^ **p**
	**No.**	**%**	**No.**	**%**		
**Pre-operative**					7.196	0.091
• Grade B	8	40.0	11	55.0		
• Grade C posterior focal	4	20.0	7	35.0		
• Grade C posterior diffuse	2	10.0	2	10.0		
• Grade C posterior subretinal	1	5.0	0	0.0		
• Grade C anterior circumferential	5	25.0	0	0.0		
**6 weeks**					4.441	0.513
• None	12	60.0	12	60.0		
• Grade A	1	5.0	0	0.0		
• Grade B	2	10.0	5	25.0		
• Grade C posterior focal	2	10.0	1	5.0		
• Grade C posterior diffuse	3	15.0	1	5.0		
• Grade C anterior circumferential	0	0.0	1	5.0		
**12 weeks**					10.024^*^	0.038^*^
• None	13	65.0	8	40.0		
• Grade B	0	0.0	6	30.0		
• Grade C posterior focal	4	20.0	2	10.0		
• Grade C posterior diffuse	3	15.0	2	10.0		
• Grade C posterior subretinal	0	0.0	1	5.0		
• Grade C anterior circumferential	0	0.0	1	5.0		
**BCVA Snellen**	**Group A**		**Group B**		**χ** ^**2**^	^**MC**^ **p**
	**No.**	**%**	**No.**	**%**		
**Pre-operative**	**(** ***n*** ** = 19)**		**(** ***n*** ** = 20)**		–	–
• HM (0.005)	19	100.0	20	100.0		
**6 weeks**	**(** ***n*** ** = 19)**		**(** ***n*** ** = 18)**		8.986	0.175
• PL (0.001)	0	0.0	1	5.6		
• HM (0.005)	7	36.8	6	33.3		
• CF 1 (0.016)	1	5.3	4	22.2		
• CF 2 (0.033)	5	26.3	6	33.3		
• CF 3 (0.05)	3	15.8	0	0.0		
• 0.1	2	10.5	0	0.0		
• 0.2	0	0.0	1	5.6		
• 0.3	1	5.3	0	0.0		
**12 weeks**	**(** ***n*** ** = 19)**		**(** ***n*** ** = 17)**		12.168^*^	0.035^*^
• PL (0.001)	0	0.0	2	11.8		
• HM (0.005)	9	47.4	5	29.4		
• CF 1 (0.016)	2	10.5	5	29.4		
• CF 2 (0.033)	0	0.0	3	17.6		
• CF 3 (0.05)	4	21.1	0	0.0		
• 0.1	2	10.5	2	11.8		
• 0.2	1	5.3	0	0.0		
• 0.4	1	5.3	0	0.0		
**Secondary procedure at 12 wk.**	**Group A (** ***n*** ** = 20)**		**Group B (** ***n*** ** = 19)**		**Test**	**p**
	**No.**	**%**	**No.**	**%**		
• None	13	65.0	15	78.9	5.212	^MC^p= 0.263
• SOR + Gas	1	5.0	0	0.0		
• SOR + PVR management + SOI	2	10.0	4	21.1		
• SOR + retinectomy + SOI	1	5.0	0	0.0		
• SOR due to high IOP	2	10.0	0	0.0		
• SOR + PVR management + SOI + phaco	1	5.0	0	0.0		
**Timing of procedure (weeks)**	**(** ***n*** ** = 7)**		**(** ***n*** ** = 4)**		U = 3.50^*^	0.042^*^
• Min.– Max.	2.0–12.0		2.0–8.0			
• Mean ± SD.	9.71 ± 3.73		4.0 ± 2.83			

SD: Standard deviation U: Mann Whitney test

χ^2^: Chi square test, FE: Fisher Exact, MC: Monte Carlo

p: p value for comparing between the two studied groups

*: Statistically significant at *p* ≤ 0.05

## Discussion

Proliferative vitreoretinopathy (PVR), especially in pediatric patients, is a major cause of failure of retinal reattachment surgery [32, 33]. It is a wound healing response resulting in the formation of a membrane on both surfaces of the retina and vitreous base [34]. Contraction of the resulting scar tissue leads to redetachment and failure of surgery. It is a complex process involving cellular proliferation of a variety of cells and secretion and remodeling of the extracellular matrix [35–37]. 

Because 5-FU and LMWH are effective in different aspects in the PVR process, it was believed that a syngergistic approach to the prevention of PVR would be advantageous.

In this study, although the rate of recurrent RRD within 12 weeks after surgery was not statistically different between treatment and control groups, treatment group showed statistically significant less advanced PVR (*p* = 0.038), later recurrences of RD (*p* = 0.042) and better final BCVA than control group (*p* = 0.035) within 12 weeks after surgery.

The beneficial effects of 5-FU and LMWH in preventing PVR in high risk RRD were also demonstrated in a randomized double-blind controlled trial of 174 high risk RRD eyes by Asaria et al. where the incidence of postoperative PVR was significantly lower (*P* = 0.02) in the 5-FU and LMWH therapy compared with the placebo group as well as rate of reoperations. The difference in visual acuity was not statistically different in the two treatment groups, although those patients in whom postoperative PVR developed tended to have poorer vision (*P* = 0.0001). There were no differences in complication rates between the two groups [38]. 

Conversely, Wickham et al. studied the effects of 5-FU and LMWH in the management of unselected RRD patients undergoing vitrectomy with the primary outcome of retinal reattachment without additional interventions at 6 months. The overall primary success rate was 84.4%; in the treatment group, the primary success rate was 82.3% compared with 86.8% in the placebo group (*P* = 0.12). At 6 months, the final complete anatomical reattachment rate was 97.9% in both treatment and placebo groups. The number of patients who failed due to the development of PVR was not statistically significant, (*P* = 0.309). There was no significant difference in the mean visual acuity at 6 months in the placebo group (0.48) versus the treatment group (0.53; *P* = 0.072) [39]. 

Interestingly, the visual acuity at 6 months of patients presenting with a macula-sparing retinal detachment was significantly worse in the treatment group (*P* = 0.0091). There was no significant difference between the 2 groups in patients who presented with a macula involving retinal detachment (*P* = 0.896) [39]. This may be because of the potentially better visual outcome in these cases compared to macula-involving detachments where the insult of photoreceptor separation may mask the toxicity of exposure to adjuvants. Likewise, in high-risk cases and established PVR, the lower levels of visual acuity seen in all cases could potentially mask a toxicity effect. So, they concluded that the combination of 5-FU and LMWH should not be routinely used in unselected primary RRD.

Sundaram et al. tried to compare both previous studies but could not perform a meta-analysis because of significant heterogeneity between them, and came to the conclusion that there is inconsistent evidence from two studies on patients at different risk of PVR on the effect of LMWH and 5-FU used during vitrectomy to prevent PVR and recommended more research to be conducted on high risk patients only, until a benefit is confirmed at least in this patient subgroup [40]. 

More recently, Schaub et al. performed an RCT to study the efficacy of intravitreal 5-FU and heparin to prevent PVR in a total of 352 eyes. The primary end point was the development of PVR grade CP (full-thickness retinal folds or subretinal strands in clock hours located posterior to equator) 1 or higher within 12 weeks after surgery. Secondary end points included best- corrected visual acuity and redetachment rate. No significant difference was found in PVR rate with odds ratio [OR], 1.25; 95% confidence interval [CI], 0.76–2.08; *P* = 0.77. None of the secondary end points showed any significant difference between treatment groups. During the study period, no relevant safety risks were identified [41]. 

In summary, pediatric RRD is still a complex surgical challenge for vitreo-retinal surgeons due to multiple factors with postoperative PVR representing one of the major obstacles to primary success. Although, we believe that evidence of the efficacy and safety of the combination of 5-FU and LMWH in preventing postoperative PVR are conflicting because of the heterogeneity in patient inclusion criteria as well as different primary and secondary end points in different studies, it may be a useful salvage therapy for children with high risk RRD.

### Limitations

- Further larger studies are needed to confirm these beneficial effects and to set criteria for eligibility as well as cut off concentrations for toxicity specific for that age group with longer follow up periods.
- Future studies should include comparison between other anti-proliferative substances e.g., methotrexate, daunorubicin and anti-VEGFs…etc. to reach a better cocktail with more solid results as regarding efficacy and safety.

## Conclusion

- Intravitreal infusion of 5-FU and LMWH can be used as an adjunct to primary vitrectomy with silicone oil tamponade and scleral buckle to prevent PVR in high risk pediatric RRD with reasonable efficacy and high degree of safety.
- This study concluded that the use of this combination in this category of patients resulted in lower rate of postoperative PVR, later recurrence of RD and better final BCVA.

## Data Availability

No datasets were generated or analysed during the current study.

## Abbreviations

5-FU: 5-fluorouracil
LMWH: Low Molecular Weight Heparin
PRRD: Pediatric rhegmatogenous retinal detachments
PVR: Proliferative vitreoretinopathy
BCVA: Best corrected visual acuity
HM: Hand movement
PL: Light perception
PVD: Posterior vitreous detachment
PPV: Pars plana vitrectomy
G: Guage
SOR: Silicone oil removal
IOP: Intraocular pressure
SD: Standard deviation
t: Student t-test
χ^2^: Chi square test
p: p value for comparing between the two studied groups
FE: Fisher Exact
U: Mann Whitney test
MC: Monte Carlo
OR: Odds ratio
CI: Confidence interval
